# Differences among Three Skeletal Muscle Mass Indices in Predicting Non-Alcoholic Fatty Liver Disease: Korean Nationwide Population-Based Study

**DOI:** 10.3390/life11080751

**Published:** 2021-07-26

**Authors:** A-Ra Cho, Jun-Hyuk Lee, Yu-Jin Kwon

**Affiliations:** 1 Department of Family Medicine, Yongin Severance Hospital, Yonsei University College of Medicine, Yongin 16995, Korea; ara1713@yuhs.ac; 2 Department of Family Medicine, Nowon Eulji Medical Center, Eulji University School of Medicine, Seoul 01830, Korea; swpapa@eulji.ac.kr

**Keywords:** non-alcoholic fatty liver, muscle mass, skeletal muscle index, sarcopenia, insulin resistance

## Abstract

Recent studies have investigated the relationship between sarcopenia and non-alcoholic fatty liver disease (NAFLD); however, there is no unified definition of sarcopenia. Thus, we aimed to investigate the differences among three skeletal muscle mass indices (SMI) in predicting NAFLD. This study included 8133 adults from the 2008–2010 Korea National Health and Nutrition Survey. SMI was calculated as appendicular skeletal muscle mass divided by height-square (hSMI), weight (wSMI), or body mass index (bSMI). The presence of NAFLD was defined by using the NAFLD-liver fat score. On the receiver operating characteristic curve analysis, the predictive power of wSMI for NAFLD was significantly higher than those of hSMI and bSMI in men (wSMI vs. hSMI, *p* = 0.003; wSMI vs. bSMI, *p* < 0.001). In women, the predictive power of hSMI was only significantly higher than that of bSMI (*p* = 0.023), and other predictive powers were not significantly different. In addition, hSMI was correlated with insulin resistance and NAFLD-liver fat score in the opposite direction to wSMI and bSMI in both men and women. Among the three definitions of SMI, wSMI showed the highest diagnostic performance for predicting NAFLD in men, suggesting the importance of defining sarcopenia for its association with specific diseases.

## 1. Introduction

Sarcopenia is a common skeletal muscle disorder, characterized by low muscle strength, low muscle quantity, and low physical performance [1]. Sarcopenia was first described as a decrease in muscle mass associated with normal aging [2]; however, it has become a serious medical problem since it is known to be associated with not only frailty, poor quality of life, and disability in the elderly [3], but also cardio-metabolic disorders [4]. The close link between sarcopenia and cardio-metabolic disorders including type 2 diabetes and cardiovascular disease has been explained by the multifactorial etiology of sarcopenia, including chronic inflammation, insulin resistance, and endocrine abnormalities [5].

Non-alcoholic fatty liver disease (NAFLD) is the leading cause of chronic liver disease worldwide. The global prevalence of NAFLD was reported to be about 25% in 2018 [6], which increased accompanied by the increasing prevalence of obesity and insulin resistance [7,8,9]. As the Westernized lifestyle and the rate of receiving health screenings have increased, the recent prevalence of NAFLD in Korea has also increased. The prevalence of NAFLD is estimated to be over 30% in Korea [10]. NAFLD ranges from simple liver steatosis to steatohepatitis, and it can progress to liver cirrhosis and liver failure [11]. The pathophysiology of NAFLD is complex, and is determined by numerous mechanisms including genetic, environmental, and metabolic factors [12]. Therefore, there has been increased interest in identifying the risk factors for NAFLD and its progression to complications [11,13].

Recent studies have investigated the relationship between sarcopenia and NAFLD because of their common pathophysiological causes such as systemic inflammation and insulin resistance [14,15,16]. However, the findings are still inconsistent, and it is also unclear whether a shortage of muscle mass, a relative excess of fat mass, or both, are associated with NAFLD.

Thus, we aimed to investigate which of the three different skeletal muscle mass indices (adjusting for height, weight, or body mass index) could predict NAFLD most accurately. Furthermore, we examined the correlations between the three skeletal muscle mass indices and both insulin resistance and NAFLD to characterize the link between sarcopenia and NAFLD via insulin resistance.

## 2. Materials and Methods

### 2.1. Study Population

We analyzed all data from the 2008–2010 Korean National Health and Nutrition Examination Survey (KNHANES). The KNHANES, annually conducted by the Korea Centers for Disease Control and Prevention (KCDC), is a nationwide, representative, and population-based survey. This survey monitors the health and nutritional status of the Korean population [17]. To represent the Korean population, participants were selected by using the proportional allocation-systematic sampling method with multistage stratification based on sex, age, and geographic area. Sampling weights were assigned to each participant to generalize the units for representing the Korean population [18]. Detailed information about the KNHANES is available through the KNHANES website (http://knhanes.cdc.go.kr, accessed on 7 June 2021). 

**Figure 1:**
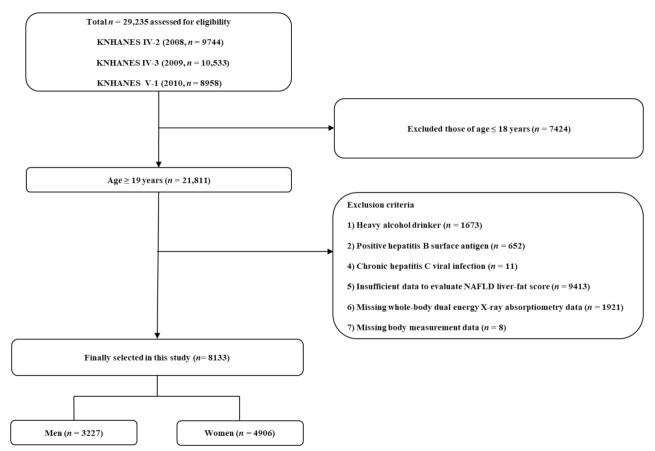
Flowchart of the study population selection.

A total of 29,235 people participated in the 2008–2010 KNHANES. We excluded 7424 participants under 19 years of age. Among the remaining 21,811 adults over 19 years old, we further excluded heavy alcoholics (*n* = 1673) and participants with positive hepatitis B surface antigen (*n* = 652); chronic hepatitis C viral infection (*n* = 11); insufficient data to evaluate NAFLD liver-fat score (*n* = 9143); missing whole-body dual-energy X-ray absorptiometry (DEXA) data (*n* = 1921); or missing height or weight measurement data (*n* = 8). Finally, a total of 8133 participants (3277 men and 4906 women) were included in the analysis (Figure 1). There were no pregnant or lactating women in the final analysis.

### 2.2. Assessment of NAFLD

NAFLD was defined by using a validated surrogate index, the NAFLD-liver fat score [19]. The calculation equation is as follows: −2.89 + 1.18 × metabolic syndrome (yes = 1/no = 0) + 0.45 × diabetes mellitus (yes = 2/no = 0) + 0.15 × fasting insulin (µU/mL) + 0.04 × aspartate aminotransferase (AST, U/L) + 0.94 × AST/alanine aminotransferase (ALT, U/L) ratio.

### 2.3. Assessment Body Composition

Height (m) and weight (kg) were measured to the nearest 0.1 cm and 0.1 kg, respectively. Body mass index (BMI) was calculated as the weight divided by height squared (kg/m^2^). A BMI less than 18.5 kg/m^2^ was considered underweight, and a BMI over 25 kg/m^2^ was considered obese according to the definition of the Korean Society for the Study of Obesity [20]. Waist circumference (cm) was measured in the horizontal plane midway between the iliac crest and the lowest rib.

During the 2008–2010 KNHANES, body composition data were collected from the head, arms, legs, trunk, pelvic region, and whole body by using whole-body DEXA (QDR 4500 A; Hologic Inc., Bedford, MA, USA). For each anatomical region, bone mineral content (g), bone mineral density (g/cm^2^), fat mass (g), lean body mass (g), and total fat percentage (fat mass/total mass × 100) were recorded. We calculated skeletal muscle mass as lean body mass (g)—bone mineral content (g). Appendicular skeletal muscle mass (ASM) was calculated by summation of skeletal muscle mass from both arms and legs. Subtotal skeletal muscle mass was calculated by summation of skeletal muscle mass from the whole body except for the head area.

### 2.4. Three Different Definitions of Skeletal Muscle Mass Index

Skeletal muscle mass index (SMI) and low skeletal muscle mass index (LSMI) were defined in three different ways. First, height square-adjusted SMI (hSMI) was ASM/height^2^ (kg/m^2^), and height square-adjusted LSMI (hLSMI) was defined as hSMI of <7.0 kg/m^2^ for men and <5.4 kg/m^2^ for women based on the Asian Working Group for Sarcopenia criteria [21]. Second, weight-adjusted SMI (wSMI) was ASM/weight × 100 (%), and weight-adjusted LSMI (wLSMI) was defined as wSMI values below −2 standard deviations of the sex-specific mean values of referent young adults aged 19–29 years [22]. Third, BMI-adjusted SMI (bSMI) was ASM/BMI (m^2^), and BMI-adjusted LSMI (bLSMI) was defined as bSMI <0.789 for men and <0.512 for women based on the Foundation for the National Institutes of Health (FNIH) Sarcopenia Project criteria [23].

### 2.5. Data Collection

Blood pressure was measured in a sitting position after at least 30 min of resting. Mean blood pressure (MBP; mmHg) was calculated using the following formula: MBP = [systolic blood pressure (SBP) + 2 × diastolic blood pressure (DBP)]/3. We defined heavy alcohol drinkers as adults who drink ≥30 g/day alcohol among men and ≥20 g/day alcohol among women. Smoking status was divided into two categories: current smoker or not. Based on the International Physical Activity Questionnaire, we defined regular exercise as ≥20 min of vigorous exercise for ≥3 days/week, or ≥30 min of moderate exercise/walking for ≥5 days/week. Each participant’s blood sample was collected from the antecubital vein after at least 8 h of fasting. Using a Hitachi 7600 Analyzer (Hitachi Co., Tokyo, Japan), plasma glucose, serum insulin, total cholesterol, triglyceride, high-density lipoprotein (HDL) cholesterol, AST and ALT levels were measured. The homeostatic assessment model of insulin resistance (HOMA-IR) was calculated following the equation: HOMA-IR = plasma glucose (mg/dL) × serum insulin (µU/mL)/405. A 24-h dietary recall method was used to assess participant’s diets. Total calorie intake (kcal/day) and percentage of protein, carbohydrates, and fat intake to total caloric intake (%) were calculated. Metabolic syndrome was defined as three or more of the following five criteria being met [24]: (1) waist circumference ≥90 cm in men or ≥85 cm in women, according to the Korean-specific cut-offs for abdominal obesity of the Korean Society of Obesity [25]; (2) SBP ≥130 mmHg, DBP ≥85 mmHg, or treatment with anti-hypertensive medications; (3) fasting plasma glucose level ≥100 mg/dL, glycosylated hemoglobin (HbA1c) ≥ 6.5%, or treatment with anti-diabetic medications; (4) serum triglyceride level ≥150 mg/dL; (5) serum HDL cholesterol level <40 mg/dL in men or <50 mg/dL in women. Based on American Diabetes Association criteria [26], diabetes mellitus was defined as either of the following: (1) fasting plasma glucose level ≥126 mg/dL, (2) HbA1c ≥ 6.5%, or treatment with anti-diabetic medications. Chronic diseases included the following six comorbid conditions: diabetes mellitus, myocardial infarction, stroke, chronic obstructive lung disease, chronic kidney disease stages from 3 to 5, and any history of cancer, considering the components of the Charlson comorbidity index [27]. Participants were categorized into three groups based on these comorbidities: zero, one, or at least two chronic diseases.

### 2.6. Statistical Analysis

We applied sampling weights to the participants to derive data representative of the Korean population. The weights were adjusted with the values for the inverse of the response rates and the inverse of the selection probability to the sex- and age-specific values for the Korean population (post-stratification) [17]. All data analyzed in this study are presented as a mean ± standard error (SE) or percentage (SE). To compare differences of continuous variables between participants with or without NAFLD, weighted generalized linear regression analysis was used. Weighted chi-square tests were used for categorical variables. Weighted logistic regression analysis was served to calculate odds ratio (OR) with 95% confidence interval (CI) for NAFLD according to different definitions of LSMI. We adjusted for age in Model 1. We adjusted for age and lifestyle (smoking status, alcohol drinking status, and regular exercise) in Model 2. We additionally adjusted for mean blood pressure, plasma glucose, serum total cholesterol, serum ALT, serum vitamin D levels and number of chronic diseases in Model 3. The receiver operating characteristics (ROC) curves were used to compare the discriminative power of wSMI, hSMI, and bSMI to predict NAFLD using the areas under the ROC curves (AUC). In addition, weighted Pearson’s correlation tests were performed to calculate correlation coefficients (r) between wSMI/hSMI/bSMI and both HOMA-IR and NAFLD-liver fat score. Steiger’s Z tests were used to compare *r* values of hSMI and bSMI with that of wSMI. All statistical analyses were conducted using SPSS statistical software (version 25.0; SPSS Inc., Chicago, IL, USA) and R (Version 4.0.3; R Foundation for Statistical Computing, Vienna, Austria). A *p* value of <0.05 was considered statistically significant.

## 3. Results

### 3.1. Clinical Characteristics of the Study Population

Table 1 represents the clinical characteristics of the study population. Participants with NAFLD showed significantly higher weight, BMI, waist circumference, MBP, plasma glucose, insulin, HOMA-IR, serum total cholesterol, AST and ALT levels, and lower proportion without chronic disease than participants without NAFLD in both men and women. In women only, the average age, percentage of carbohydrate intake, and percentage of fat intake were significantly higher, and height, the proportion of alcohol drinkers and those who exercise regularly were significantly lower in participants with NAFLD. Only in men, the proportion of never smokers was significantly lower in participants with NAFLD. In both men and women, NAFLD-liver fat score, ASM, and hSMI were significantly higher in people with NAFLD, whereas wSMI and bSMI were significantly lower in those with NAFLD.

### 3.2. Comparison of Body Composition according to Three Different Definitions of LSMI

Table 2 shows body composition according to three different definitions of LSMI. The mean values of ASM and subtotal skeletal muscle mass were significantly lower, and percentage of appendicular fat was significantly higher in participants with sarcopenia in all three definitions. However, appendicular fat mass, subtotal fat mass, and percentage of subtotal fat mass were significantly higher in sarcopenia defined by wSMI and bSMI, whereas appendicular fat mass and subtotal fat mass were lower in sarcopenia defined by hSMI, in both men and women.

### 3.3. Relationship between Three Different Definitions of LSMI and NAFLD

Table 3 represents the results of weighted logistic regression analysis showing the relationship between three different definitions of LSMI and NAFLD. The unadjusted ORs (95% CI) for NAFLD of wLSMI/hLSMI/bLSMI were 3.39 (1.92–5.97), 0.55 (0.41–0.74) and 1.88 (1.36–2.59) in men and 2.37 (1.62–3.48), 0.38 (0.29–0.50) and 2.12 (1.59–2.84) in women, respectively. After adjusting for age, smoking status, alcohol drinking status, regular exercise, mean blood pressure, serum total cholesterol, serum vitamin D levels, number of chronic diseases, and percentage of protein intake, the adjusted ORs (95% CI) for NAFLD of wLSMI/hLSMI/bLSMI were 2.52 (1.20–5.30), 0.50 (0.36–0.71), and 1.55 (1.03–2.33) in men and 1.98 (1.34–2.93), 0.48 (0.35–0.65), and 1.30 (0.92–1.85) in women, respectively.

### 3.4. Comparison of the Predictive Power of Three Different Definitions of SMI for NAFLD

**Figure 2:**
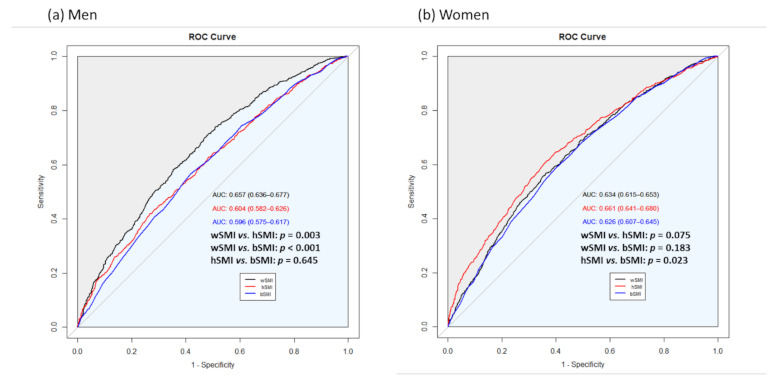
Comparison of the predictive power of three different definitions of SMI for NAFLD in (**a**) men and (**b**) women.

Figure 2 compares the predictive power of wSMI, hSMI, and bSMI for NAFLD. The AUC of wSMI, hSMI, and bSMI were 0.657, 0.604, and 0.596 in men and 0.634, 0.661, and 0.626 in women, respectively. In men, despite the relatively low AUC, we found that the predictive power was highest in wSMI, followed by hSMI and bSMI (wSMI vs. hSMI, *p* = 0.003; wSMI vs. bSMI, *p* < 0.001). There was no significant difference in the predictive power between hSMI and bSMI (*p* = 0.645). In women, the predictive power of hSMI was significantly higher than that of bSMI (hSMI vs. bSMI, *p* = 0.023); however, there were no differences in the predictive power between wSMI and hSMI, bSMI (wSMI vs. hSMI, *p* = 0.075; wSMI vs. bSMI, *p* = 0.183).

### 3.5. Correlations between Three Different Definitions of SMI and HOMA-IR/NAFLD

Table 4 shows a comparison of r values between three definitions of SMI and HOMA-IR. The r values between wSMI/hSMI/bSMI and HOMA-IR were −0.318, 0.192, and −0.212 in men and −0.214, 0.290, and −0.184 in women, respectively. In men, the absolute value of r of wSMI was significantly higher than that of hSMI or bSMI (wSMI vs. hSMI, *p* value < 0.001; wSMI vs. bSMI, *p* < 0.001). In women, the absolute value of r of wSMI was significantly lower than that of hSMI (*p* < 0.001) and higher than that of bSMI (*p* < 0.001).

Table 5 shows a comparison of r values between three definitions of SMI and NAFLD-liver fat score; r values between wSMI/hSMI/bSMI and NAFLD-liver fat score were −0.283, 0.224, and −0.182 in men and −0.237, 0.302, and −0.232 in women, respectively. In men, the absolute value of r of wSMI was significantly higher than those of hSMI or bSMI (wSMI vs. hSMI, *p* = 0.004; wSMI vs. bSMI, *p* < 0.001). In women, the absolute value of r of wSMI was significantly lower than that of hSMI (wSMI vs. hSMI, *p* < 0.001), whereas there was no significant difference between the absolute value of r of wSMI and bSMI (wSMI vs. bSMI, *p* = 0.542).

## 4. Discussion

We examined the predictive power of three different definitions of skeletal muscle mass indices for NAFLD in a representative sample of Korean adults. Our results show that the predictive power of wSMI was significantly higher than those of hSMI and bSMI in men. The predictive power of hSMI was only significantly higher than that of bSMI in women; however, there were no significant differences between wSMI and hSMI or bSMI in women. In addition, wSMI and bSMI were negatively correlated with HOMA-IR and NAFLD-liver fat score, whereas hSMI was positively correlated in both men and women.

Many expert groups have suggested operational criteria for sarcopenia using the different anthropometric indices (e.g., weight-adjusted, height square-adjusted, and BMI-adjusted indices) [1,21,23]. Many epidemiologic studies have used these different operational definitions to investigate the association between sarcopenia and various pathologic conditions (e.g., cardio-metabolic diseases, recurrent falling and fracture, and mortality). However, the observed prevalence of sarcopenia using the different definitions was very different and agreement among the three definitions was also very low [28]. Several meta-analysis studies found significant associations between sarcopenia and NAFLD/liver fibrosis [15,29]; however, these studies did not consider the different criteria for sarcopenia. In 2014, Hong et al. [30] suggested a significant relationship between sarcopenia defined by wSMI and NAFLD after adjusting for potential confounding factors. Lee et al. [31] reported that sarcopenia defined by wSMI is associated with increased risks of NAFLD and advanced fibrosis, independent of obesity or metabolic syndrome using the same cohort dataset used in our study. However, Peng’s study conducted in 2019 [16] demonstrated that for sarcopenia defined by hSMI, its association with NAFLD is in the opposite direction to wSMI. They first suggested that the different definitions of SMI could substantially influence study outcomes, particularly in relation to NAFLD. Peng’s study used a bio-resistance body composition analyzer for assessing muscle mass and they conducted the study only in participants aged 60–75 years [16]. Most recently, Kang et al. [32] examined the association of skeletal muscle and adipose tissue distribution with biopsy-proven NAFLD. Kang’s study defined sarcopenia by hSMI and showed a negative association between hLSMI and hepatic steatosis.

The results of our study are consistent with previous studies suggesting that different sarcopenia definitions have different associations with NAFLD. In the current study, we found a positive association between wLSMI/bLSMI and NAFLD and a negative association between hLSMI and NAFLD in adults aged 19 years and older using sex-specific analysis.

Possible mechanisms of the interaction between sarcopenia and NAFLD are based on effects of insulin resistance [33,34], chronic inflammation [30], and crosstalk between organs by secretion of cytokines, such as hepatokines, adipokines, and myokines [35,36]. The present study showed a significantly higher correlation between wSMI and both HOMA-IR and NAFLD-liver fat score in men, whereas hSMI showed a correlation in the opposite direction in both men and women. These results suggest that the link between sarcopenia and NAFLD is via insulin resistance. In addition, we compared body composition according to the three definitions of SMI to determine the relative fat mass associated with low muscle mass in each definition. Interestingly, appendicular fat mass, subtotal fat mass, and percentage of subtotal fat mass were significantly higher in sarcopenia defined by wSMI and bSMI, whereas appendicular fat mass and subtotal fat mass were lower in sarcopenia defined by hSMI. When muscle mass was adjusted by weight and BMI, subjects with sarcopenia may have high relative fat mass, which could be the main cause of the association with NAFLD.

Although the reason that the predictive power of hSMI was only significantly higher than that of bSMI, and others were not significantly different in women, is unclear, sex-based differences in skeletal muscle fiber type, composition and function hormonal effects might affect these discrepant results.

This study also has several limitations. First, we could not examine muscle strength and physical performance due to lack of data in the KNHANES. Since sarcopenia is defined as a decrease in muscle mass, muscle strength, and physical performance, further studies are needed to confirm the predictive power for NAFLD according to muscle strength and physical performance as well as muscle mass. Second, we used a validated non-invasive biomarker, NAFLD-liver fat score, to assess NAFLD, rather than liver imaging or histological information. The gold standard for diagnosis of NAFLD is liver biopsy; however, it has disadvantages such as high cost, sampling error, and complications related to invasive procedures [37]. Finally, the causal relationship between low muscle mass and NAFLD could not be demonstrated because of the cross-sectional study design. Further longitudinal studies are required to find the causal relationship between sarcopenia and NAFLD.

The strength and novelty of our study is that we not only compare the predictive power of the three definitions of SMI for NAFLD, but also demonstrate the possible explanations for the results by showing the association with insulin resistance and the comparison of fat mass. Second, our data assessed muscle mass using the DXA, which is a gold standard tool for measurement of body composition, including muscle mass. Finally, we conducted a sex-specific analysis considering the different body compositions of men and women. Although there is still no unified definition of LSMI, our study showed the highest diagnostic performance of wSMI for the prediction of NAFLD in men.

## 5. Conclusions

The predictive power of wSMI for NAFLD is significantly higher than those of hSMI and bSMI in men. Moreover, hSMI shows correlations with HOMA-IR and NAFLD-liver fat score in the opposite direction to wSMI/bSMI. Consensus on the operational criteria to clarify the association between sarcopenia and NAFLD is much needed.

## Figures and Tables

**Table 1:** Clinical characteristics of the study population.

	Men			Women		
Variables	Without NAFLD	With NAFLD	*p*	Without NAFLD	With NAFLD	*p*
Unweighted number, *n*	2311	916		3920	986	
Age, years	48.6 ± 0.5	47.3 ± 0.8	0.153	46.6 ± 0.4	54.5 ± 0.7	<0.001
Height, m	1.698 ± 0.002	1.702 ± 0.004	0.356	1.572 ± 0.002	1.555 ± 0.003	<0.001
Weight, kg	67.4 ± 0.3	74.4 ± 0.6	<0.001	56.1 ± 0.2	62.0 ± 0.5	<0.001
BMI, kg/m^2^	23.3 ± 0.1	25.6 ± 0.2	<0.001	22.7 ± 0.1	25.6 ± 0.2	<0.001
Waist circumference, cm	82.3 ± 0.3	88.8 ± 0.4	<0.001	76.3 ± 0.3	85.2 ± 0.5	<0.001
Mean blood pressure, mmHg	93.2 ± 0.3	95.4 ± 0.5	0.001	87.5 ± 0.4	93.6 ± 0.6	<0.001
Smoking status, % (SE)			0.030			0.134
Never smoker	21.7 (1.1)	17.2 (1.6)		90.0 (0.7)	92.0 (1.3)	
Ex-smoker	13.9 (1.2)	17.9 (2.0)		1.4 (0.3)	0.7 (0.2)	
Current smoker	64.4 (1.5)	64.9 (2.3)		8.6 (0.6)	7.3 (1.3)	
Alcohol drinker, % (SE)	73.1 (1.4)	69.7 (2.1)	0.151	39.8 (1.1)	34.4 (2.1)	0.033
Regular exercise, % (SE)	27.6 (1.4)	24.5 (1.7)	0.145	21.2 (1.0)	16.9 (1.8)	<0.001
Glucose, mg/dL	95.7 ± 0.4	108.3 ± 1.2	<0.001	92.5 ± 0.3	110.0 ± 1.3	<0.001
Insulin, µIU/mLt	8.3 ± 0.1	13.7 ± 0.3	<0.001	8.7 ± 0.1	14.4 ± 0.3	<0.001
HOMA-IR	2.0 ± 0.0	3.7 ± 0.1	<0.001	2.0 ± 0.0	3.9 ± 0.1	<0.001
Total cholesterol, mg/dL	186.1 ± 0.9	191.5 ± 1.7	0.006	185.5 ± 0.8	196.0 ± 1.7	<0.001
AST, U/L	21.5 ± 0.2	32.7 ± 1.4	<0.001	18.7 ± 0.1	26.8 ± 0.7	<0.001
ALT, U/L	20.8 ± 0.3	42.9 ± 1.3	<0.001	14.9 ± 0.1	29.1 ± 0.9	<0.001
Total caloric intake, kcal/day	2228.9 ± 28.6	2222.2 ± 39.7	0.879	1653.8 ± 14.8	1595.7 ± 33.3	0.096
Protein intake, %	14.5 ± 0.1	14.7 ± 0.2	0.458	14.2 ± 0.1	14.1 ± 0.2	0.441
Carbohydrate intake, %	65.3 ± 0.4	64.9 ± 0.7	0.562	69.5 ± 0.3	71.2 ± 0.6	0.005
Fat intake, %	17.2 ± 0.3	17.7 ± 0.5	0.301	16.7 ± 0.2	15.6 ± 0.4	0.028
Number of chronic diseases, % (SE)			<0.001			<0.001
0	86.34 (1.0)	64.7 (2.2		89.8 (0.7)	60.0 (2.3)	
1	10.7 (0.9)	27.6 (2.1)		9.0 (0.7)	32.4 (2.2)	
≥2	2.9 (0.5)	7.7 (1.5		1.2 (0.3)	7.6 (1.3)	
NAFLD-liver fat score	−1.672 ± 0.018	0.465 ± 0.056	<0.001	−1.915 ± 0.015	0.257 ± 0.039	<0.001
ASM, kg	21.9 ± 0.1	23.0 ± 0.2	<0.001	14.3 ± 0.1	15.0 ± 0.1	<0.001
wSMI, %	32.6 ± 0.1	31.0 ± 0.1	<0.001	25.5 ± 0.1	24.3 ± 0.1	<0.001
hSMI, kg/m^2^	7.6 ± 0.0	7.9 ± 0.1	<0.001	5.8 ± 0.0	6.2 ± 0.0	<0.001
bSMI, m^2^	0.944 ± 0.004	0.902 ± 0.006	<0.001	0.640 ± 0.003	0.590 ± 0.004	<0.001

*p* values were derived from weighted generalized linear regression analysis for continuous variables and weighted chi-square tests for categorical variables. Abbreviations: NAFLD, non-alcoholic fatty liver disease; BMI, body mass index; HOMA-IR, homeostatic model assessment of insulin resistance; AST, aspartate aminotransferase; ALT, alanine aminotransferase; ASM, appendicular skeletal muscle mass; wSMI, weight-adjusted skeletal muscle mass index; hSMI, height-adjusted skeletal muscle mass index; bSMI, body mass index-adjusted skeletal muscle mass index; SE, standard error.

**Table 2:** Body composition according to three different definitions of LSMI.

	LSMI Defined by wSMI			LSMI Defined by hSMI			LSMI Defined by bSMI		
	No	Yes	*p*	No	Yes	*p*	No	Yes	*p*
Men									
Unweighted number, *n*	3106	121		2463	764		2851	376	
Appendicular skeletal muscle mass, kg	22.3 ± 0.1	19.1 ± 0.4	<0.001	23.3 ± 0.1	18.5 ± 0.1	<0.001	22.6 ± 0.1	18.5 ± 0.2	<0.001
Appendicular fat mass, kg	5.9 ± 0.0	8.9 ± 0.2	<0.001	6.2 ± 0.0	5.2 ± 0.1	<0.001	5.9 ± 0.0	6.7 ± 0.2	<0.001
Percentage of appendicular fat, %	19.6 ± 0.2	30.3 ± 0.5	<0.001	19.8 ± 0.2	20.7 ± 0.4	0.020	19.4 ± 0.2	25.0 ± 0.4	<0.001
Subtotal skeletal muscle mass, kg *	46.9 ± 0.2	42.9 ± 1.0	<0.001	48.7 ± 0.2	39.8 ± 0.2	<0.001	47.4 ± 0.2	40.9 ± 0.4	<0.001
Subtotal fat mass, kg	14.2 ± 0.1	22.8 ± 0.6	<0.001	15.1 ± 0.2	12.5 ± 0.3	<0.001	14.2 ± 0.2	17.4 ± 0.4	<0.001
Percentage of subtotal fat, %	22.1 ± 0.2	33.6 ± 0.4	<0.001	22.5 ± 0.2	22.6 ± 0.4	0.667	21.8 ± 0.2	28.6 ± 0.4	<0.001
Women									
Unweighted number, *n*	4677	229		3518	1388		4397	509	
Appendicular skeletal muscle mass, kg	14.5 ± 0.0	12.6 ± 0.3	<0.001	15.1 ± 0.0	12.5 ± 0.1	<0.001	14.6 ± 0.0	12.3 ± 0.1	<0.001
Appendicular fat mass, kg	8.4 ± 0.0	11.4 ± 0.3	<0.001	8.8 ± 0.0	7.9 ± 0.0	<0.001	8.5 ± 0.0	9.4 ± 0.2	<0.001
Percentage of appendicular fat, %	35.2 ± 0.1	45.4 ± 0.8	<0.001	35.2 ± 0.1	36.8 ± 0.3	<0.001	35.0 ± 0.1	41.3 ± 0.6	<0.001
Subtotal skeletal muscle mass, kg *	32.5 ± 0.1	30.4 ± 0.6	0.001	33.8 ± 0.1	28.8 ± 0.1	<0.001	32.8 ± 0.1	29.4 ± 0.3	<0.001
Subtotal fat mass, kg	17.8 ± 0.1	26.2 ± 0.5	<0.001	19.1 ± 0.1	15.8 ± 0.2	<0.001	17.8 ± 0.1	22.0 ± 0.4	<0.001
Percentage of subtotal fat, %	34.0 ± 0.1	45.0 ± 0.6	<0.001	34.7 ± 0.2	34.0 ± 0.3	0.044	33.8 ± 0.1	41.5 ± 0.5	<0.001

wSMI was defined as appendicular skeletal muscle mass divided by weight (%); hSMI index was defined as appendicular skeletal muscle mass divided by height square (kg/m^2^); bSMI was defined as appendicular skeletal muscle mass divided by body mass index (m^2^). * subtotal area includes whole body except the head area. Abbreviations: LSMI, low skeletal muscle mass index; wSMI, weight-adjusted skeletal muscle mass index; hSMI, height-adjusted skeletal muscle mass index; bSMI, body mass index-adjusted skeletal muscle mass index.

**Table 3:** Weighted logistic regression analysis showing relationships between three different definitions of LSMI and NAFLD.

	Men			Women		
	NAFLD			NAFLD		
	No	Yes		No	Yes	
		OR (95% CI)	*p*		OR (95% CI)	*p*
LSMI defined by wSMI						
Unadjusted	1 (Ref.)	3.39 (1.92–5.97)	<0.001	1 (Ref.)	2.37 (1.62–3.48)	<0.001
Model 1	1 (Ref.)	3.67 (2.08–6.47)	<0.001	1 (Ref.)	1.83 (1.27–2.62)	0.001
Model 2	1 (Ref.)	3.61(2.09–6.26)	<0.001	1 (Ref.)	1.82 (1.27–2.63)	0.001
Model 3	1 (Ref.)	2.52 (1.20–5.30)	0.015	1 (Ref.)	1.98 (1.34–2.93)	0.001
LSMI defined by hSMI						
Unadjusted	1 (Ref.)	0.55 (0.41–0.74)	<0.001	1 (Ref.)	0.38 (0.29–0.50)	<0.001
Model 1	1 (Ref.)	0.56 (0.42–0.75)	<0.001	1 (Ref.)	0.44 (0.34–0.57)	<0.001
Model 2	1 (Ref.)	0.55 (0.41–0.74)	<0.001	1 (Ref.)	0.43 (0.33–0.56)	<0.001
Model 3	1 (Ref.)	0.50 (0.36–0.71)	<0.001	1 (Ref.)	0.48 (0.35–0.65)	<0.001
LSMI defined by bSMI						
Unadjusted	1 (Ref.)	1.88 (1.36–2.59)	<0.001	1 (Ref.)	2.12 (1.59–2.84)	<0.001
Model 1	1 (Ref.)	2.15 (1.53–3.02)	<0.001	1 (Ref.)	1.35 (0.99–1.84)	0.056
Model 2	1 (Ref.)	2.06 (1.46–2.90)	<0.001	1 (Ref.)	1.36 (1.00–1.85)	0.054
Model 3	1 (Ref.)	1.55 (1.03–2.33)	0.001	1 (Ref.)	1.30 (0.92–1.85)	0.141

Model 1: Adjusted for age; Model 2: Adjusted for age, smoking status, alcohol drinking status, and regular exercise; Model 3: Adjusted for age, smoking status, alcohol drinking status, regular exercise, mean blood pressure, serum total cholesterol, serum vitamin D levels, number of chronic diseases, and percentage of protein intake. Abbreviations: LSMI, low skeletal muscle mass index; wSMI, weight-adjusted skeletal muscle mass index; hSMI, height-adjusted skeletal muscle mass index; bSMI, body mass index-adjusted skeletal muscle mass index; NAFLD, non-alcoholic fatty liver disease; OR, odds ratio; CI, confidence interval.

**Table 4:** Comparison of correlation coefficients (r) between three different definitions of SMI and HOMA-IR.

		*r*	*p* *	*p* **
Men	wSMI	−0.318	<0.001	Ref.
	hSMI	0.192	<0.001	<0.001
	bSMI	−0.212	<0.001	<0.001
Women	wSMI	−0.214	<0.001	Ref.
	hSMI	0.290	<0.001	<0.001
	bSMI	−0.184	<0.001	<0.001

* *p* for r between wSMI/hSMI/bSMI and HOMA-IR. ** *p* indicates *p* values for comparison of absolute values of r using Steiger’s Z-test. Abbreviations: wSMI, weight-adjusted skeletal muscle mass index; hSMI, height-adjusted skeletal muscle mass index; bSMI, body mass index-adjusted skeletal muscle mass index; HOMA-IR, homeostatic model assessment of insulin resistance.

**Table 5:** Comparison of correlation coefficients (r) between three different definitions of SMI and NAFLD-liver fat score.

		*r*	*p* *	*p* **
Men	wSMI	−0.283	<0.001	Ref
	hSMI	0.224	<0.001	0.004
	bSMI	−0.182	<0.001	<0.001
Women	wSMI	−0.237	<0.001	Ref
	hSMI	0.302	<0.001	<0.001
	bSMI	−0.232	<0.001	0.542

* *p* for r between wSMI/hSMI/bSMI and NAFLD-liver fat score. ** *p* indicates *p* values for comparison of absolute values of r using Steiger’s Z-test. Abbreviations: wSMI, weight-adjusted skeletal muscle mass index; hSMI, height-adjusted skeletal muscle mass index; bSMI, body mass index-adjusted skeletal muscle mass index; NAFLD, non-alcoholic fatty liver disease.

## Data Availability

The KNHANES data are publicly available through the KNHANES website (https://knhanes.kdca.go.kr/knhanes, accessed on 7 June 2021).
